# Impact of intraoperative stimulation mapping on high-grade glioma surgery outcome: a meta-analysis

**DOI:** 10.1007/s00701-018-3732-4

**Published:** 2018-11-21

**Authors:** Jasper Kees Wim Gerritsen, Lidia Arends, Markus Klimek, Clemens Maria Franciscus Dirven, Arnaud Jean-Pierre Edouard Vincent

**Affiliations:** 1 Department of Neurosurgery, Erasmus Medical Center Rotterdam, ‘s-Gravendijkwal 230, 3015 CE Rotterdam, The Netherlands; 2 Department of Biostatistics, Erasmus Medical Center Rotterdam, Rotterdam, The Netherlands; 3 Department of Anesthesiology, Erasmus Medical Center Rotterdam, Rotterdam, The Netherlands

**Keywords:** Awake craniotomy, Glioblastoma, Extent of resection, Morbidity, Mortality

## Abstract

**Background:**

Intraoperative stimulation mapping (ISM) using electrocortical mapping (awake craniotomy, AC) or evoked potentials has become a solid option for the resection of supratentorial low-grade gliomas in eloquent areas, but not as much for high-grade gliomas. This meta-analysis aims to determine whether the surgeon, when using ISM and AC, is able to achieve improved overall survival and decreased neurological morbidity in patients with high-grade glioma as compared to resection under general anesthesia (GA).

**Methods:**

A systematic search was performed to identify relevant studies. Adult patients were included who had undergone craniotomy for high-grade glioma (WHO grade III or IV) using ISM (among which AC) or GA. Primary outcomes were rate of postoperative complications, overall postoperative survival, and percentage of gross total resections (GTR). Secondary outcomes were extent of resection and percentage of eloquent areas.

**Results:**

Review of 2049 articles led to the inclusion of 53 studies in the analysis, including 9102 patients. The overall postoperative median survival in the AC group was significantly longer (16.87 versus 12.04 months; *p* < 0.001) and the postoperative complication rate was significantly lower (0.13 versus 0.21; *p* < 0.001). Mean percentage of GTR was significantly higher in the ISM group (79.1% versus 47.7%, *p* < 0.0001). Extent of resection and preoperative patient KPS were indicated as prognostic factors, whereas patient KPS and involvement of eloquent areas were identified as predictive factors.

**Conclusions:**

These findings suggest that surgeons using ISM and AC during their resections of high-grade glioma in eloquent areas experienced better surgical outcomes: a significantly longer overall postoperative survival, a lower rate of postoperative complications, and a higher percentage of GTR.

**Electronic supplementary material:**

The online version of this article (10.1007/s00701-018-3732-4) contains supplementary material, which is available to authorized users.

## Introduction

Glioblastomas (WHO IV glioma) are devastating tumors with one of the worst prognoses in oncology. The median survival after surgery and combined treatment with chemo- and radiotherapy ranges from 12 to 15 months and no curative therapy is currently available [8, 23]. Multiple studies show that extent of resection of the contrast enhancing part of the tumor improves survival in patients with GBM [15–18, 20–22, 24]. Further analyses showed that patients who previously had complete resections derived the most benefit from the temozolomide (TMZ) regimen compared with those who had had incomplete resection [1]. Thus, in addition to the survival benefit associated with maximum cytoreductive surgery, such surgery seems essential for the efficacy of modern adjuvant treatment. More than 50% of GBMs are located near or in eloquent areas of the brain. Damaging these areas during surgery can lead to severe and permanent neurological deficits that seriously impact the quality of life. Therefore, when resecting GBMs in these areas, they are usually not operated as aggressive as possible, due to the chance of seriously damaging the patient with a rather low life expectancy [13, 15–18, 20, 23]. However, patients with partial or subtotal resections will benefit less from radio- and chemotherapy as compared to patients with total resections [15–18, 20–22, 24].Intraoperative stimulation mapping (ISM) allows the surgeon to prevent damage to eloquent cortical and subcortical areas during resection [2, 19]. There is compelling evidence that surgeons using ISM experience increased resection percentage while preserving quality of life in low-grade glioma (LGG). We expect that the use of awake craniotomy by surgeons therefore is also of important value in the surgery of GBM, and in a similar fashion can optimize the extent of resection and preserve quality of life, thereby improving survival in these patients [2, 5–7, 9–12, 14, 19].

The usefullness of ISM by surgeons and its impact on neurologic outcome has been evaluated mainly for mostly low-grade gliomas or as a descriptive review. In this article, a meta-analysis is performed to compare the surgeon’s use of intraoperative stimulation mapping (among which awake craniotomy, AC) versus general anesthesia for the resection of high-grade glioma.

## Methods

### Search strategy

A computer-aided search of Embase, Medline (OvidSP), Web of Science, the Cochrane Library, Pubmed, and Google Scholar was performed to identify relevant studies. The search terms used were (craniotomy OR surgery OR surgical approach OR surgical patient OR surgical technique OR brain surgery OR brain tumor OR cancer surgery OR neurosurgery OR intraoperative period) AND (wakefulness OR sedation OR conscious sedation OR consciousness OR arousal OR local anesthesia OR local anesthetic agent OR electrostimulation OR sensorimotor function OR (stimulation AND brain cortex)) AND (glioma OR brain tumor OR brain cancer OR intracranial tumor OR gliobastoma OR ((brain OR intracranial OR supratentorial OR cortex OR cortical) NEAR (tumor OR tumour OR cancer OR lesion)) NOT (conference abstract OR letter OR note OR editorial) AND (english). The publication period was restricted to January 1, 1990, to April 1, 2017. One reviewer (JKWG) performed the initial search in association with a biomedical information specialist of the library service of Erasmus Medical Centre, who verified the search. Reference lists of the studies were searched for additional valuable studies.

### Study selection criteria

**Fig. 1:**
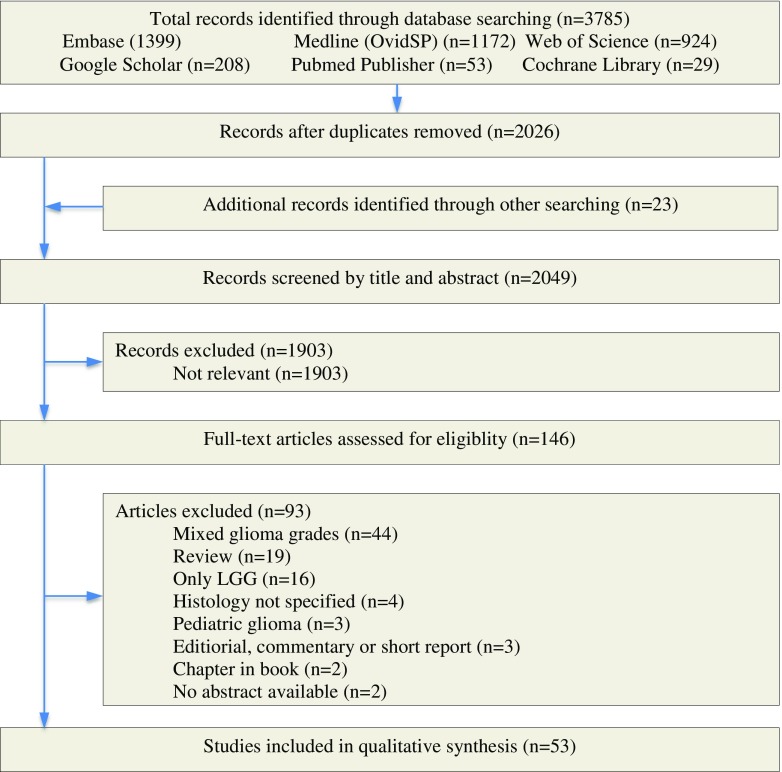
Flowchart of total records identified through database searching

To be included in the meta-analysis, all studies had to have examined the effects of resective glioma surgery with or without the use of ISM by surgeons. Studies were reviewed that used resective glioma surgery to improve the prognosis in patients with high-grade glioma (WHO III-IV). Only complemented studies meeting the PICO format of this study where full-text versions were available were included. PICO (Population Intervention Comparison Outcome) restrictions were made for population (patients under 18 years old were excluded), intervention (fMRI, DTI, MSI, neuronavigation, or ultrasound were not considered ISM), and outcome: eligible primary outcomes were survival, extent of resection (percentage gross total resection—GTR), and complication rate. Studies were exluded if they included patients with glioma grading other than WHO grade 3 or 4; patients under 18 years old; when the pathohistology of the tumors was not specified; when the article was of review-, editorial-, commentary-, short report-format or was a chapter in a book; and when no abstract was available (Fig. 1, flowchart).

### Outcome measures and definitions

The primary outcome measures were the event rate of postoperative complications, overall survival, and percentage GTR. Postoperative complications were noted as such as defined by colleagues de Witt Hamer et al. [7] Complications were not categorized according to severity and timing of assessment. Complications were eligible as such when they emerged postoperatively; when pre-operative symptoms worsened; or when they were part of a worsening of the patients’ condition postoperatively. The percentage of patients in whom GTR was obtained according to postoperative neuroimaging was also extracted. Furthermore, data regarding patient KPS and the percentage of craniotomies concerning eloquent areas was collected. Sources related to publication, population, or management characteristics were distinguished. Publication-related characteristics were publication year, continent, and study setting. Patient population-related characteristics were mean age and percentage of eloquently located gliomas. Treatment-related characteristics were intraoperative techniques and sort of anesthesia (awake or general anesthesia). Intraoperative techniques included percentage of patients with resections using ISM. For ISM, electrocortical mapping was distinguished from motor- or somatosensory evoked potentials (MEP, SEP).

### Statistical analysis

Differences between the ISM group (with or without AC) and GA group for the primary outcomes were tested: (1) overall postoperative survival and (2) rate of postoperative complications. Analysis of the the data set for primary outcomes was based on non-parametrics tests; for number of complications, the Mann-Whitney test was used, whereas for survival, the log-rank test was used and for the difference in percentage GTR between groups, a two-tailed *t* test. No adjustment for multiple testing has been done. The significance level was set to 5%. Forest plots were made with SPSS (Version 24.0; IBM Analytics). Analysis of the relationships between factors was done using a mixed effects regression analysis (unrestricted ML).

## Results

### Study characteristics

The search strategy yielded 3785 publications, of which 2026 remained after duplicates were removed. Twenty-three additional recoreds were identified through alternative search strategies, mainly by searching the reference lists of the studies, increasing the total number of records identified to 2049. Following screening by title and abstract, 146 articles were considered relevant and were assessed for eligibility. Review of these 146 articles led to the inclusion of 53 studies in the analysis, including 9102 patients. A total of 1260 patients were operated using ISM; 7842 patients were operated under general anesthesia. The study characteristics of the 53 publications are listed in the Data Supplement. Not all studies allowed extraction of all end points. The complications mainly consisted of motor and language deficits (Data Supplement). The cohorts varied between 9 and 1229 patients. Included articles were publicated between 1999 and 2016. Twenty-two articles were of European origin, 21 articles were of North-American origin, eight articles were of Asian origin, two articles were of South-American origin, and one article was of Middle-East origin. Forty-eight studies were performed in a university setting (91%). Eleven studies used electrostimulation (sub) cortical mapping intraoperatively. Eleven studies used evoked potentials (such as MEPs, SEPs) intraoperatively. Four studies used awake craniotomy as anesthetic modality. The mean age of the study populations differed between 49.0 and 78.0 years. Percentage of eloquently located gliomas differed between 0 and 100%. The percentage of patients in whom GTR was obtained differed between 6 and 96%. The overall postoperative median survival of patients following diagnosis differed between 4.5 and 16.3 months. Postoperative complication rates differed between 0.0 and 0.64.

### Overall survival

**Fig. 2:**
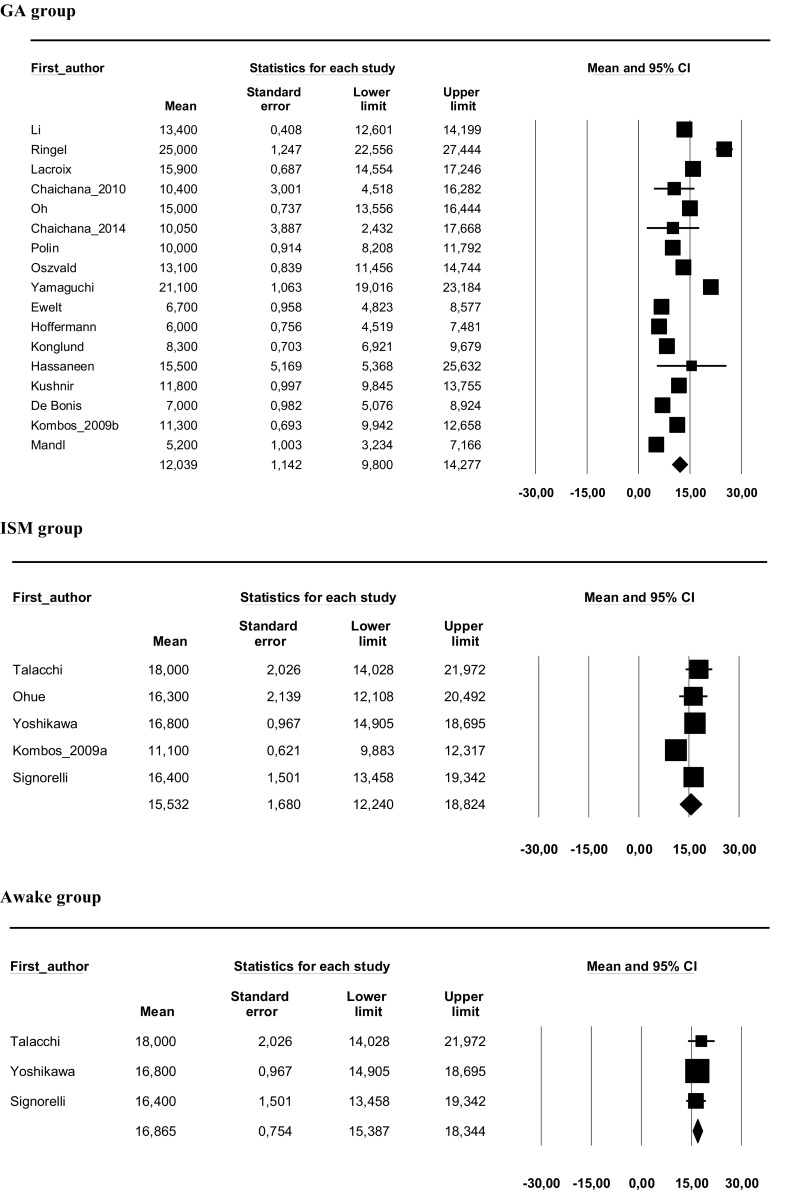
Forest plot

The median overall survival rates for each study are provided in Table 1 of the Data Supplement. Studies evaluating craniotomy under GA with data on overall survival (*n* = 17) included 4390 patients with a median overall survival of 12.04 months (SE = 1.14; 95% CI 9.80–14.28). Studies evaluating craniotomy with ISM with available data (*n* = 5) included 279 patients with a median overall survival of 15.53 months (SE = 1.68; 95% CI 12.24–18.82). Studies evaluating awake craniotomy (subgroep of ISM) with available data (*n* = 3) included 210 patients with a median overall survival of 16.87 months (SE = 0.75; 95% CI 15.40–18.34). The median survival in the ISM group was almost 3.5 months longer than in the GA group, but this was not significant (*p* = 0.085). The median survival in the awake group was more than 4.5 months longer than in the GA group, which was significant (*p* < 0.001). Forest plots for median overall survival rates are displayed in Fig. 2.

### Extent of resection and survival

**Fig. 3:**
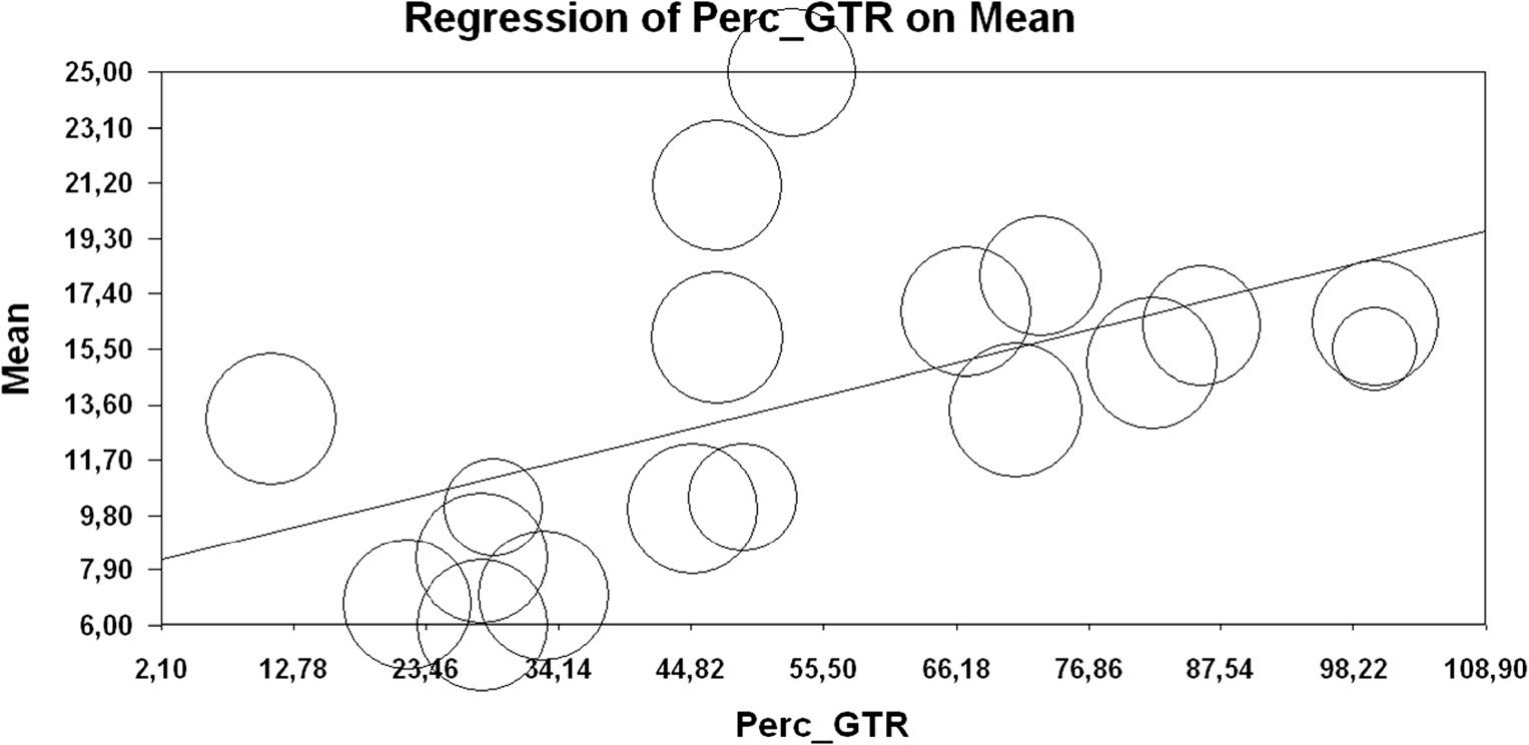
Extent of resection and overall survival

Eighteen studies evaluated the extent of resection (expressed as percentage of craniotomies in which gross total resection (GTR) was achieved) in correlation with overall survival. Percentages of craniotomies in which GTR was achieved in each study are provided in Table 1 of the Data Supplement. Using a mixed effects regression with unrestricted ML, a significant positive relation was found between extent of resection and overall survival (*b* = 0.11; SE = 0.04; *p* = 0.012) (Fig. 3), indicating extent of resection as a major prognostic factor in high-grade glioma surgery.

### Preoperative patient KPS and survival

Forty-five studies evaluated preoperative patient KPS (Karnofsky Performance Score) in correlation with overall survival. Preoperative patient KPS are provided in Table 1 of the Data Supplement. Using a mixed effects regression with unrestricted ML, a significant positive relation was found between preoperative patient KPS and overall survival (*b* = 0.61; SE = 0.13; *p* < 0.001), indicating preoperative patient KPS as a major prognostic factor in high-grade glioma surgery.

### Postoperative complications

The postoperative complication rates for each study are provided in Table 1 of the Data Supplement. Studies evaluating craniotomy under GA with data on postoperative complications (*n* = 19) included 5826 patients with a total of 1250 postoperative complications. In this group, the postoperative complication rate was 0.21 (95% CI 0.20–0.23). Studies evaluating craniotomy with ISM with available data (*n* = 9) included 430 patients with a total of 54 postoperative complications. In this group, the postoperative complication rate was 0.13 (95% CI 0.10–0.16). The complication rate in the ISM group was significantly lower than in the GA group (*p* < 0.001).

### Extent of resection

The extent of resection is expressed as percentage of GTR obtained for each study. The data are provided in Table 1 of the Data Supplement. Studies evaluating craniotomy under GA with data on extent of resection (*n* = 24) included 6880 patients. In 3283 cases, GTR was obtained. In this group, the mean percentage of GTR was 47.7% (95% CI 40.4–55.5). Studies evaluating craniotomy with ISM with available data (*n* = 6) included 369 patients. In 292 cases, GTR was obtained. In this group, the mean percentage of GTR was 79.1% (95% CI 69.8–88.4). The mean percentage of GTR in the ISM group was significantly higher than in the GA group (*p* < 0.001).

### Extent of resection and postoperative complications

**Fig. 4:**
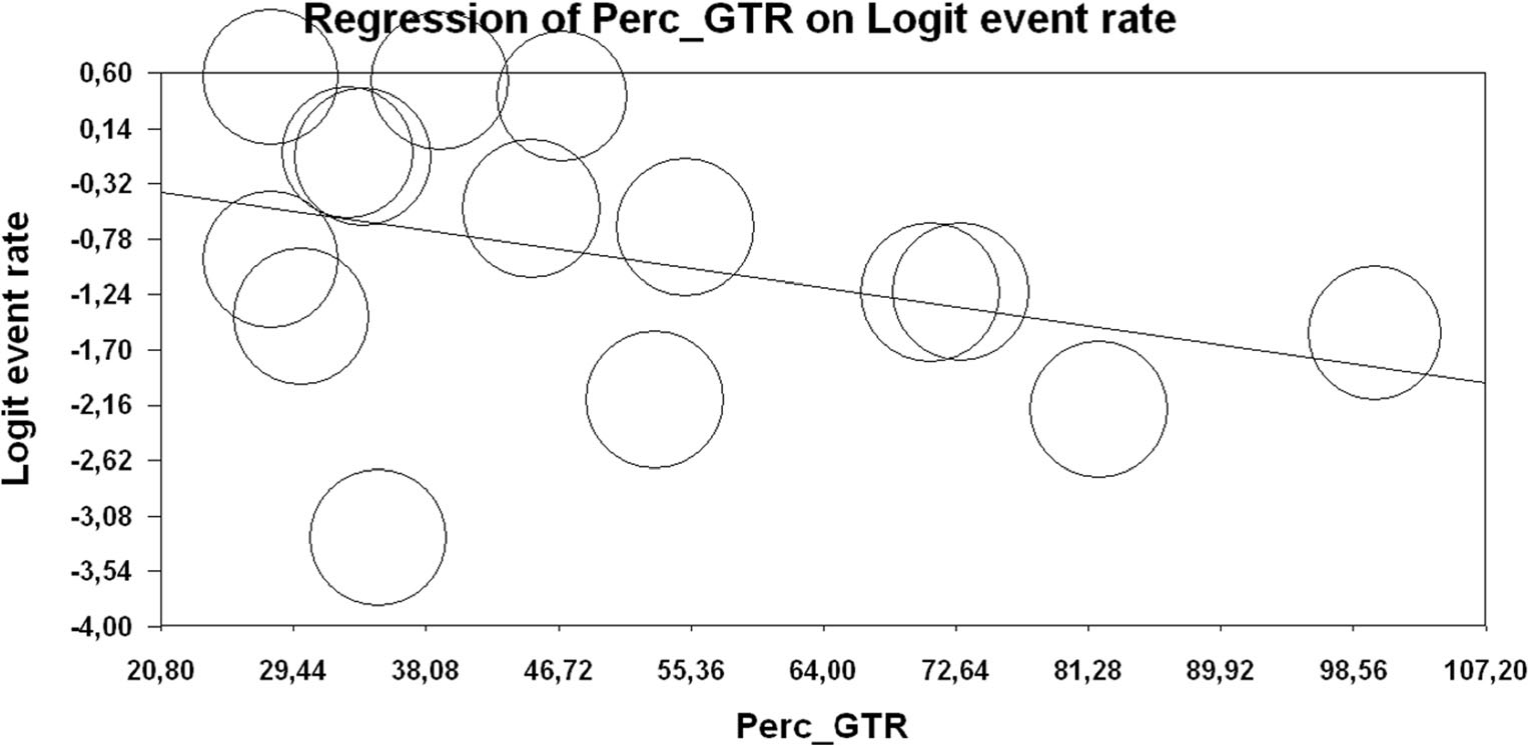
Extent of resection and postoperative complication rate

Fifteen studies evaluated extent of resection (expressed as percentage of craniotomies in which gross total resection (GTR) was achieved) in correlation with postoperative complications. Percentages of craniotomies in which GTR was achieved in each study are provided in Table 1 of the Data Supplement. Using a mixed effects regression with unrestricted ML, no relation was found between extent of resection and overall survival (*b* = − 0.018; SE-0.012; *p* = 0.132) (Fig. 4), indicating that achieving a higher extent of resection does not yield a higher rate of postoperative complications.

### Patient KPS and postoperative complications

Ten studies evaluated preoperative patient KPS in correlation with postoperative complications. Percentages of eloquent areas in each study are provided in Table 1 of the Data Supplement. Using a mixed effects regression with unrestricted ML, a significant positive relation was found between patient KPS and postoperative complications (*b* = − 0.095; SE = 0.039; *p* = 0.014), indicating preoperative patient KPS as a major predictive factor for postoperative complications in high-grade glioma surgery.

### Eloquent areas and postoperative complications

Eleven studies evaluated the percentage of eloquent areas in correlation with postoperative complications. Percentages of eloquent areas in each study are provided in Table 1 of the Data Supplement. Using a mixed effects regression with unrestricted ML, no overall relation was found between the percentage of eloquent areas and postoperative complications (*b* = − 0.009; SE = 0.013; *p* = 0.475). However, a significant relation was found when evaluating only studies investigating craniotomies under GA, indicating that a higher percentage of eloquent areas was significantly positively related with a higher postoperative complication rate (*b* = 0.044; SE = 0.007; *p* < 0.001).

## Discussion

This meta-analysis shows that patients who had been operated by surgeons using AC as ISM for a single supratentorial high-grade glioma had a significant longer overall postoperative median survival (more than 4.5 months longer) and were subject of less postoperative complications in eloquent areas (0.13 versus 0.21). Furthermore, the percentage of resections in which GTR was obtained was significantly higher in the ISM group as compared to the GA group (47.7% versus 79.1, *p* < 0.001). The use of ISM and AC by surgeons is safe, as a greater extent of resection did not yield a higher rate of complications. Moreover, extent of resection and preoperative patient KPS were indicated as prognostic factors, whereas patient KPS and the involvement of eloquent areas were identified as predictive factors. These results suggest that the use of ISM (AC in particular) by surgeons should be implemented as a routine operation for surgery of high-grade tumors near eloquent areas of the brain.

It is important to recognize the ifs and buts of the use of adjunct surgical techniques such as ISM and AC. The results yielded by the use of such techniques are only as good as the surgeon who uses these techniques. The fact that no technique can ever replace knowledge, experience and skill should be acknowledged, valued, and acted upon accordingly. Mapping and monitoring during glioma resections are useless if the surgeon is not familiar with using these techniques. Implementing new techniques takes practice and will inevitably come with a certain learning curve regarding both technical use and case selection.

Neurosurgeons have a daunting task: resecting the tumor with an extent as great as possible, while simultaneously minimizing the risk for postoperative complications and especially neurological morbidity. Surgeons use ISM to maximize resection, primarily to increase the patient’s survival while minimizing the chances of morbidity and loss of neurological function [2, 24]. AC is the most frequently used form of ISM, by using electrocortical and subcortical mapping to differ eloquent brain tissue from brain or tumor tissue that is safe to resect. Hereby, surgeons try to maximize the extent of resection with at the same time minimizing the risk of postoperative complications. To date, surgeons use ISM and AC in particular for the resection of low-grade gliomas because of the usually near-eloquent location of these tumors [4, 13]. Only few studies have evaluated the use of these techniques in high-grade gliomas, as is reflected in the studies included (see also Table 1, Data Supplement). We showed that surgeons using AC can significantly contribute to this goal by preserving the quality of life of these patients and decreasing the risk of postoperative morbidity when operating in eloquent areas, while increasing extent of resection and maximizing postoperative survival.

To the best of our knowledge, this is the first study that systematically investigates the use of ISM and AC by surgeons in high-grade glioma surgery only.

A study and meta-analysis very similar to ours, conducted by De Witt Hamer et al., included 8091 patients with supratentorial infiltrative glioma (high- and low-grade glioma) that were resected by surgeons using ISM or not [7]. They found that glioma resections in which the surgeon had used ISM were associated with fewer late major neurologic deficits. These findings are in accordance with our results evaluating high-grade glioma resections, since we found that surgeons using AC as ISM experienced decreased rates of postoperative complications in eloquent areas.

Sacko et al. prospectively compared surgeons using AC versus craniotomy under GA for resection of supratentorial lesions including 575 glioma patients [19]. They found that patients with tumors in eloquent areas revealed a significantly better neurological outcome and extent of resection in the AC group than the GA group. Although this study also includes low-grade glioma patients, it is one of the largest prospective studies comparing surgeons using AC and craniotomy under GA head-to-head for postoperative outcomes in glioma surgery. We found similar results after our data analysis, suggesting a role for the use of AC by surgeons in resections for high-grade glioma—especially in eloquent areas—to improve outcomes after craniotomies.

Chaichana et al. conducted a retrospective study at the Johns Hopkins University to develop a prognostic grading system in glioblastoma patients [3]. They found that (among others) a poor preoperative performance status proved to be a strong prognostic factor in glioblastoma surgery. In accordance with this findings, we found that preoperative patient KPS was not only indicated as a prognostic factor, but also a predictive factor (a poor preoperative KPS indicating an increased risk of postoperative complications). These results underline the importance of identifying subgroups of patients within the high-grade glioma patient population and the role ISM/AC use by surgeons can play in optimalization of surgery outcomes.

This study should be interpreted whitin the limitations of a meta-analysis based on observational studies. The selected publications are observational or retrospective in nature and therefore subject to selection bias, publication bias, and subjective outcome assesments, as mentioned before by de Witt et al. [7]. We therefore advise a randomized controlled trial where awake craniotomy with ISM is compared to surgery under general anesthesia for GBM near eloquent areas. Primary outcomes should be focused on neurological morbidity and extent of resection.

Publication bias can result in our findings being rather precise, with small 95% CIs, but less accurate as an estimation of the ground truth of outcome in general neurosurgical practice, since published data does not necessarily reflect general clinical practice. For example, the overall low rate of mortality is known to correlate with large-volume centers. We therefore think our results may apply to high-grade glioma patients treated in large-volume academic hospitals dedicated to neuro-oncology. Secondly, the selection bias of our findings can be expected due to patient selection with various indications for surgical intervention. However, we minimized the risk for this bias by our vast amount of data and number of included studies of patients.

## Conclusions

Surgeons resecting high-grade glioma with ISM are able to achieve a higher percentage of GTR, and the use of AC by surgeons is associated with significantly longer overall postoperative survival with less postoperative complications as compared with craniotomy under GA. The greater extent of resection achieved by mapping techniques did not yield a higher rate of complications. Furthermore, extent of resection and preoperative patient KPS were indicated as prognostic factors, whereas patient KPS and the involvement of eloquent areas were identified as predictive factors. Our findings confirm preliminary findings of other authors with smaller group sizes and elaborate on large studies with both low-grade and high-grade patient cohorts. Future studies should focus on evaluating the role of the use of AC by surgeons in the treatment of high-grade glioma and optimize risk stratification using prognostic factors. Subgroups of patients should be identified that might benefit the most from extensive surgery and AC. If future studies confirm and elaborate on the results presented in this study, the role of awake craniotomies in neurooncology should be revisited and expanded.

## Electronic supplementary material

ESM 1 (DOCX 149 kb)
