# Supplementary material for: Impact of intraoperative stimulation mapping on high-grade glioma surgery outcome: a meta-analysis
Source: Acta Neurochir (Wien). 2018 Nov 21;161(1):99–107. doi: 10.1007/s00701-018-3732-4 (PMC6331492; doi:10.1007/s00701-018-3732-4)
Supplement: Supplementary file 1 — (DOCX 149 kb) [file 701_2018_3732_MOESM1_ESM.docx]

| **First author** | **Year** | **Mean age** | **Electro-stimulation (ISM)** | **Evoked potentials (ISM)** | **Conventional (GA)** | **Awake (AC/**  **ISM)** | **% eloquent**  **areas** | **% GTR** | **Median survival (mo) (95%CI)** | **Complications** | **Median KPS pre-op** |
| --- | --- | --- | --- | --- | --- | --- | --- | --- | --- | --- | --- |
| Li | 2016 | 55.7 | 0 | 0 | 1 | 0 | 38 | 71 | 13.4 (12.6-14.1) | 279/1229 | 90 |
| McGirt | 2009 | 51.0 | 0 | 0 | 1 | 0 | NA | 35 | 12.3 (NA) | 34/949 | 80 |
| Chaichana | 2009 | 55.0 | 1 | 1 | 1 | 0 | NA | 33 | NA | 63/648 | 80 |
| Ringel | 2016 | 57.0 | 0 | 0 | 1 | 0 | NA | 53 | 25 (22.5-27.4) | 54/503 | 90 |
| Sanai | 2011 | 60.0 | 0 | 0 | 1 | 0 | NA | 69 | 12.2 (NA) | NA/500 | 80 |
| Lacroix | 2001 | 53.0 | 0 | 0 | 1 | 0 | 44 | 47 | 15.9 (14.6-17.3) | NA/416 | 80 |
| Chaichana | 2010 | 58.6 | 0 | 0 | 1 | 0 | 28 | 49 | 10.4 (4.7-16.5) | NA/393 | 80 |
| McGirt | 2009 | 54.0 | 0 | 0 | 1 | 0 | NA | 55 | 12.8 (NA) | 103/306 | 80 |
| Oh | 2014 | 52.0 | 0 | 0 | 1 | 0 | NA | 82 | 15.0 (13.6-16.5) | 30/301 | 80 |
| Chaichana | 2014 | 62.0 | 0 | 0 | 1 | 0 | 29 | 29 | 12.6 (2.4-17.7) | NA/292 | 90 |
| Polin | 2005 | 60.0 | 0 | 0 | 1 | 0 | 50 | 45 | 10 (9.0-11.8) | 104/280 | 80 |
| Ening | 2015 | NA | 0 | 0 | 1 | 0 | 78 | 39 | NA | 159/252 | 70 |
| D’Amico | 2015 | 73.0 | 0 | 0 | 1 | 0 | NA | NA | 9.0 (NA) | 52/243 | 80 |
| Oszvald | 2012 | 61.0 | 0 | 0 | 1 | 0 | NA | 11 | 13.1 (±12.9) | NA/234 | 80 |
| Talacchi | 2010 | NA | 1 | 1 | 0 | 1 | 100 | 73 | 18 (15.0-23.0) | 39/171 | 80 |
| Yamaguchi | 2012 | 55.0 | 0 | 0 | 1 | 0 | 56 | 47 | 21.1 (19-23.2) | NA/160 | 80 |
| Gulati | 2011 | 62.0 | 0 | 0 | 1 | 0 | NA | 34 | NA | 67/141 | 70 |
| Martinez | 2007 | 58.3 | 1 | 1 | 1 | 0 | 67 | 82 | 6.0 (±4.4) | 76/138 | 70 |
| Chaichana | 2011 | 73.0 | 0 | 0 | 1 | 0 | 25 | 30 | 7.9 (NA) | 25/129 | 80 |
| Grabowski | 2014 | 60.0 | 0 | 0 | 1 | 0 | 24 | 6 | 13.8 (NA) | NA/128 | 90 |
| Dea | 2012 | 60.5 | 0 | 0 | 1 | 0 | NA | NA | 8.9 (NA) | NA/126 | NA |
| Uzuka | 2012 | 65.0 | 0 | 0 | 1 | 0 | NA | NA | 13.5 (NA) | NA/107 | 60 |
| Ewelt | 2011 | 70.8 | 0 | 0 | 1 | 0 | NA | 22 | 6.7 (4.8-8.1) | NA/103 | 70 |
| Lorenzoni | 2008 | 49.0 | 0 | 0 | 1 | 0 | NA | NA | 12.0 (NA) | 12/103 | NA |
| Hoffermann | 2015 | 71.0 | 0 | 0 | 1 | 0 | NA | 28 | 6.0 (4.5-7.5) | 27/97 | 70 |
| Scott | 2011 | 76.1 | 0 | 0 | 1 | 0 | NA | 25 | 4.5 (NA) | NA/93 | NA |
| Keles | 1999 | 51.0 | 0 | 0 | 1 | 0 | NA | 25 | 14.0 (NA) | NA/92 | 90 |
| Shinoda | 2001 | 57.4 | 0 | 0 | 1 | 0 | 44 | 44 | 13.0 (NA) | 9/82 | 60 |
| Konglund | 2013 | 68.5 | 0 | 0 | 1 | 0 | 59 | 28 | 8.3 (6.9-9.7) | 51/80 | 80 |
| Uzuka | 2014 | 78.0 | 0 | 0 | 1 | 0 | NA | NA | 9.8 (NA) | 27/79 | 60 |
| Hassaneen | 2011 | NA | 0 | 0 | 1 | 0 | NA | 100 | 9.7 (5.2-25.8) | 13/75 | 80 |
| Marina | 2011 | 69.0 | 0 | 0 | 1 | 0 | NA | 20 | 5.8 (NA) | NA/74 | 50 |
| Muacevic | 2003 | 69.4 | 0 | 0 | 1 | 0 | NA | NA | 5.5 (NA) | 3/58 | 70 |
| Benveniste | 2005 | 54.9 | 1 | 1 | 0 | 0 | NA | 87 | NA | 2/54 | NA |
| Schucht | 2012 | 60.0 | 1 | 1 | 0 | 0 | 100 | 96 | 16.7 (NA) | 2/53 | 80 |
| Ulmer | 2006 | 58.0 | 0 | 0 | 1 | 0 | NA | NA | NA | 20/50 | NA |
| Ohue | 2015 | 64.7 | 1 | 1 | 0 | 0 | 100 | 86 | 16.3 (12.0-22.5) | NA/49 | 80 |
| Orringer | 2012 | NA | 0 | 0 | 1 | 0 | 39 | 17 | NA | NA/46 | NA |
| Kushnir | 2011 | 71.9 | 0 | 0 | 1 | 0 | NA | NA | 11.8 (±6.66) | NA/42 | NA |
| Reithmeier | 2003 | NA | 0 | 1 | 0 | 0 | 100 | NA | NA | 6/42 | NA |
| Pastor | 2013 | 49.8 | 1 | 1 | 0 | 0 | 100 | 67 | NA | 1/34 | 70 |
| De Bonis | 2013 | 59.0 | 0 | 0 | 1 | 0 | NA | 33 | 7.0 (5.0-9.0) | 16/33 | 70 |
| Kurimoto | 2007 | 73.0 | 0 | 0 | 1 | 0 | 62 | 47 | 10.5 (NA) | 18/30 | 70 |
| Pontes | 2013 | 73.0 | 0 | 0 | 1 | 0 | NA | 27 | 10.6 (NA) | NA/30 | 70 |
| Bogosaljevic | 2011 | 55.4 | 1 | 0 | 0 | 0 | 100 | NA | NA | NA/26 | 80 |
| Yoshikawa | 2006 | 61.4 | 1 | 1 | 0 | 1 | 100 | 67 | 16.8 (14.8-18.8) | 2/24 | 80 |
| Kombos | 2009 | NA | 0 | 1 | 0 | 0 | 100 | NA | 11.1 (9.8-12.4) | 0/20 | 80 |
| Kombos | 2009 | NA | 0 | 0 | 1 | 0 | 0 | NA | 11.3 (9.8-12.7) | NA/20 | 80 |
| Mandl | 2008 | 52.0 | 0 | 0 | 1 | 0 | NA | NA | 5.2 (3.1-7.3) | 7/20 | 70 |
| Feigl | 2010 | 55.0 | 0 | 1 | 0 | 0 | 100 | 64 | 11.0 (NA) | NA/18 | 90 |
| Pirracchio | 2010 | 73.5 | 0 | 0 | 1 | 0 | NA | NA | NA | NA/17 | 80 |
| Spena | 2013 | 53.7 | 1 | 0 | 0 | 1 | 100 | NA | NA | 0/17 | NA |
| Signorelli | 2001 | 57.5 | 1 | 0 | 0 | 1 | 100 | NA | 16.4 (±6.36) | 2/15 | 80 |
| Tanaka | 2012 | 73.0 | 0 | 0 | 1 | 0 | 45 | NA | 12.0 (NA) | 1/9 | 80 |

**Data supplement for**

**Impact of intraoperative stimulation mapping on high-grade glioma surgery outcome: A meta-analysis**

J.K.W. Gerritsen, L.R. Arends, M. Klimek, C.M.F. Dirven, A.J.P.E. Vincent

**Table 1: Study characteristics**

**Table 1 continued: Study characteristics**

| **First author** | **Year** | **PMID reference** | **Country** | **University setting** |
| --- | --- | --- | --- | --- |
| Li | 2016 | 26495941 J Neurosurg 2016;124:977-988 | United States | 1 |
| McGirt | 2009 | 18847342 Neurosurgery 2009;65:463-470s | United States | 1 |
| Chaichana | 2009 | 19344222 J Neurosurg 2009;111:282-92 | United States | 1 |
| Ringel | 2016 | 26243790 Neuro-Oncology 2016;18:96-104 | Germany | 1 |
| Sanai | 2011 | 21417701 J Neurosurg 2011;115:3-8 | United States | 1 |
| Lacroix | 2001 | 11780887 J Neurosurg 2001;95:190-198 | United States | 1 |
| Chaichana | 2010 | 19817542 J Neurosurg 2010;112:997-1004 | United States | 1 |
| McGirt | 2009 | 19687690 Neurosurgery 2009;65:463-9 | United States | 1 |
| Oh | 2014 | 24553726 Acta Neurochir 2014;156:641-51 | South Korea | 1 |
| Chaichana | 2014 | 24508595 World Neurosurg 2014;82:e257-65 | United States | 1 |
| Polin | 2005 | 15739555 J Neurosurg 2005;102:276-283 | United States | 1 |
| Ening | 2015 | 25942630 Clin Neurol Neurosurg 2015;134:55-9 | Germany | 1 |
| D’Amico | 2015 | 26074434 World Neurosurg 2015;4:913-9 | United States | 1 |
| Oszvald | 2012 | 21942727 J Neurosurg 2012;116:357-64 | Germany | 1 |
| Talacchi | 2010 | 20467787 J Neurooncol 2010;100:417-26 | Italy | 1 |
| Gulati | 2011 | 22251506 World Neurosurg 2011;76:572-9 | Norway | 1 |
| Martinez | 2007 | 17963194 Zentralbl Neurochir 2007;68:176-81 | Germany | 1 |
| Chaichana | 2011 | 20887095 J Neurosurg 2011;114:587-94 | United States | 1 |
| Yamaguchi | 2012 | 22399670 Jpn J Clin Oncol 2012;42:270-7 | Japan | 1 |
| Grabowski | 2014 | 25192475 J Neurosurg 2014;121:1115-1123 | United States | 1 |
| Dea | 2012 | 22931705 Can J Neurol Sci 2012;39:632-7 | Canada | 1 |
| Uzuka | 2012 | 22976140 Neurol Med Chir (Tokyo) 2012;52:570-6 | Japan | 1 |
| Ewelt | 2011 | 20953662 J Neurooncol 2011;103:611-8 | Germany | 1 |
| Lorenzoni | 2008 | 18440602 Surg Neurol 2008;70:591-7 | Chili | 1 |
| Hoffermann | 2015 | 25462098 Clin Neurol Neurosurg 2015;128:60-9 | Austria | 1 |
| Scott | 2011 | 21363881 Neuro Oncol 2011;13:428-36 | United States | 1 |
| Keles | 1999 | 10555843 Surg Neurol 1999;52:371-9 | United States | 1 |
| Shinoda | 2001 | 11508816 J Neurooncol 2001;52:161-71 | Japan | 1 |
| Konglund | 2013 | 23432636 Acta Neurol Scand 2013;128:185-93 | Norway | 1 |
| Uzuka | 2014 | 24173683 J Neurooncol 2014;116:299-306 | Japan | 1 |
| Hassaneen | 2011 | 20690813 J Neurosurg 2011;114:576-584 | United States | 1 |
| Marina | 2011 | 21548745 J Neurosurg 2011; 115:220-9 | United States | 1 |
| Muacevic | 2003 | 12736735 J Neurol 2003;250:561-8 | Germany | 1 |
| Benveniste | 2005 | 15936381 Surg Neurol 2005;63:542-8 | United States | 1 |
| Schucht | 2012 | 22895402 Neurosurgery 2012;71:927-36 | Switzerland | 1 |
| Ulmer | 2006 | 17101902 Neurology 2006;67:1668-70 | United States | 1 |
| Ohue | 2015 | 25403686 Neurosurg Rev 2015;38:293-307 | Japan | 1 |
| Orringer | 2012 | 22978537 J Neurosurg 2012;117:851-9 | United States | 1 |
| Kushnir | 2011 | 21845970 Isr Med Assoc J 2011;13:290-4 | Israel | 0 |
| Reithmeier | 2003 | 12761674 Minim Invas Neurosurg 2003;46:65-71 | Germany | 1 |
| Pastor | 2013 | 24072425 Acta Neurochir 2013;155:2201-13 | Spain | 1 |
| De Bonis | 2013 | 22959214 Clin Neurol Neurosurg 2013;115:883-6 | Italy | 1 |
| Kurimoto | 2007 | 18159138 Neurol Med Chir (Tokyo) 2007;47:543-9 | Japan | 1 |
| Pontes | 2013 | 24472484 J Geriatr Oncol 2013;4:388-93 | Brazil | 1 |
| Bogosaljevic | 2011 | 22437285 Turkish Neurosurgery 2012;22:135-40 | Serbia | 0 |
| Yoshikawa | 2006 | 16314936 Journal of Neuro-Oncology 2006;78:91-7 | Japan | 1 |
| Kombos | 2009 | 19952567 J Clin Neurophysiol 2009;26:422-5 | Germany | 1 |
| Kombos | 2009 | 19952567 J Clin Neurophysiol 2009;26:422-5 | Germany | 1 |
| Mandl | 2008 | 18262245 Surg Neurol 2008;69:506-9 | Netherlands | 1 |
| Feigl | 2010 | 19911888 J Neurosurg 2010;113:352-7 | Germany | 1 |
| Pirracchio | 2010 | 20622683 J Neurosurg Anesthesiol 2010;22:342-6 | France | 1 |
| Spena | 2013 | 23465617 Clin Neurol Neurosurg 2013;115:1595-1601 | Italy | 1 |
| Signorelli | 2001 | 11487189 Neurol Sci 2001;22:3-10 | France | 0 |
| Tanaka | 2012 | 22875708 J Neurooncol 2012;110:227-35 | United States | 1 |
